# Genetic and pharmacologic p32-inhibition rescue CHCHD2-linked Parkinson’s disease phenotypes in vivo and in cell models

**DOI:** 10.1186/s12929-024-01010-z

**Published:** 2024-02-23

**Authors:** Murni Tio, Rujing Wen, Cai Ning Choo, Jian Bin Tan, Aaron Chua, Bin Xiao, Jeyapriya Rajameenakshi Sundaram, Christine Hui Shan Chan, Eng-King Tan

**Affiliations:** 1 Department of Neurology, National Neuroscience Institute, Singapore, Singapore; 2 Department of Neurology, Singapore General Hospital, Singapore, Singapore; 3 Duke-NUS Graduate Medical School, Singapore, Singapore

**Keywords:** Parkinson’s disease, CHCHD2, p32, *Drosophila*, Isogenic line, p32-inhibition, Mitochondria

## Abstract

**Background:**

Mutations in *CHCHD2* have been linked to Parkinson’s disease, however, their exact pathophysiologic roles are unclear. The p32 protein has been suggested to interact with CHCHD2, however, the physiological functions of such interaction in the context of PD have not been clarified.

**Methods:**

Interaction between CHCHD2 and p32 was confirmed by co-immunoprecipitation experiments. We studied the effect of p32-knockdown in the transgenic *Drosophila* and Hela cells expressing the wild type and the pathogenic variants of hCHCHD2. We further investigated the rescue ability of a custom generated p32-inhibitor in these models as well as in the human fibroblast derived neural precursor cells and the dopaminergic neurons harboring *hCHCHD2-Arg145Gln*.

**Results:**

Our results showed that wildtype and mutant hCHCHD2 could bind to p32 in vitro, supported by in vivo interaction between human CHCHD2 and *Drosophila* p32. Knockdown of p32 reduced mutant hCHCHD2 levels in *Drosophila* and in vitro. In *Drosophila hCHCHD2* models, inhibition of p32 through genetic knockdown and pharmacological treatment using a customized p32-inhibitor restored dopaminergic neuron numbers and improved mitochondrial morphology. These were correlated with improved locomotor function, reduced oxidative stress and decreased mortality. Consistently, Hela cells expressing mutant hCHCHD2 showed improved mitochondrial morphology and function after treatment with the p32-inhibitor. As compared to the isogenic control cells, large percentage of the mutant neural precursor cells and dopaminergic neurons harboring *hCHCHD2-Arg145Gln* contained fragmented mitochondria which was accompanied by lower ATP production and cell viability. The NPCs harboring *hCHCHD2-Arg145Gln* also had a marked increase in α-synuclein expression. The p32-inhibitor was able to ameliorate the mitochondrial fragmentation, restored ATP levels, increased cell viability and reduced α-synuclein level in these cells.

**Conclusions:**

Our study identified p32 as a modulator of CHCHD2, possibly exerting its effects by reducing the toxic mutant hCHCHD2 expression and/or mitigating the downstream effects. Inhibition of the p32 pathway can be a potential therapeutic intervention for CHCHD2-linked PD and diseases involving mitochondrial dysfunction.

**Supplementary Information:**

The online version contains supplementary material available at 10.1186/s12929-024-01010-z.

## Background

Parkinson’s disease (PD) is a chronic, progressive and age-dependent neurodegenerative disorder characterized by selective loss of dopaminergic neurons in the substantia nigra of the brain [1, 2]. Abnormal accumulation and aggregation of α-synuclein are part of the characteristics of PD [3]. PD affects around 1% of the world population of age > 60 years old [4]. The degeneration of dopaminergic neurons in PD is accompanied by motor symptoms [5], non-motor symptoms [6], and physical defects which can cause significant morbidity and mortality [7]. Studies of PD genetic variants have shed light on the affected mechanisms involving mitochondrial function [8, 9], ubiquitination [10], vesicle transport and recycling [11], oxidative stress [12], and mitophagy [13, 14]. The modes of inheritance of familial PD can be autosomal-dominant (e.g. *alpha-synuclein*/*SNCA* and *leucine-rich repeat kinase 2*/*LRRK2* genes) [15, 16] or autosomal-recessive (e.g. *PARK2*/*Parkin*, *PARK7*/*DJ1* and *PTEN-induced kinase 1/PINK1* genes) [17–19].

Genetic variants in the *coiled-coil-helix-coiled-coil-helix domain containing 2* (*CHCHD2*) were reported predominantly in the Asian populations and with autosomal-dominant inheritance. The two pathogenic missense mutations in the *CHCHD2* gene (c.182C > T, p.Thr61Ile and c.434G > A, p.Arg145Gln) were initially identified in the Japanese population [20], and later also found in the Chinese population [21, 22]. Reported rare exonic CHCHD2 variants include the risk variant in the Asian population (P2L) [22–24], a nonsense mutation [25], and a homozygous missense mutation [26] identified in the Caucasian populations. CHCHD2 localizes in the mitochondrial inner membrane where it is responsible for maintaining oxidative phosphorylation [27], and regulating apoptosis [28, 29]. p32-CHCHD2 interaction has been suggested based on affinity purification, mass spectrometry, sucrose gradient sedimentation and co-immunoprecipitation assays [30, 31]. However, the physiological functions of the interaction between CHCHD2 and p32 in the context of PD have not been clarified [28, 32]. The p32 protein mainly localizes in the mitochondrial matrix and plays a role in maintaining oxidative phosphorylation [33–35]. Association of p32 with the inner mitochondrial membrane and its localization outside of the mitochondria provides an explanation for the diverse roles of p32 in RNA splicing, cell polarity determination and synaptic transmission [36–41]. The p32 protein is known to bind ARF causing ARF to localize to the mitochondria and to trigger apoptosis [42]. Cell surface localization of p32 in adipocytes and some cancer cells were found to play key roles in lamellipodia formation and cancer metastasis [43].

Mitochondria are the machineries for generating adenosine triphosphate (ATP), which is needed for cell survival. Mitochondria are dynamic organelles which undergo cycles of fission and fusion (called “mitochondrial dynamics”) [44]. These two events must be balanced in order for mitochondria to function properly and they are controlled by a group of mitochondrial-shaping proteins such as optic atrophy-1/OPA-1, Mitofusion-1/2 which promote fusion and dynamin-related protein-1/DRP-1, mitochondrial fission protein-1, mitochondrial fission factor, mitochondrial dynamics proteins of 49 and 51 kDa, which promote fission. Abnormal mitochondrial dynamics cause mitochondrial dysfunction and have been implicated in the disease pathogenesis of many neurodegenerative diseases [45–47].

*Drosophila* melanogaster provides a powerful genetic and in vivo model system for the study of human neurodegenerative diseases. Due to the presence of relatively complex nervous systems with organizational similarities to the vertebrate brain and conservation of genes/gene functions [48], studies of human neurodegenerative diseases in *Drosophila* have successfully recapitulated the human disease characteristics and pathogenesis [49, 50]. We previously reported that overexpression of mutant hCHCHD2 caused degeneration of dopaminergic neurons, mitochondrial dysfunction and higher level of oxidative stress which were accompanied by age-dependent locomotor deficit and shortened lifespan [51].

The clustered regularly interspaced short palindromic repeats (CRISPR)/Cas9 genome editing technology has been widely used to generate isogenic human disease models [52]. Improvement in the induced pluripotent stem cells (iPSCs) technology makes it possible to model human diseases in a relevant cellular context [53]. Neural stem cells (NSCs)/neural progenitor cells (NPCs) and the functional mature cells differentiated from the embryonic stem cells or somatic cells have been successfully used to model human diseases which shed light on the affected disease mechanisms [54, 55].

Here, we examined the CHCHD2-p32 interaction in PD *Drosophila* transgenic models and showed that knockdown of p32 was able to mitigate PD-phenotypes, possibly by reducing the toxic mutant hCHCHD2 expression and/or mitigation of the downstream phenotypes. The effects of p32 knockdown were supported by the ability of the p32-inhibitor (p32-I) treatment in ameliorating the phenotypic abnormalities in transgenic *Drosophila* expressing the pathogenic variants of hCHCHD2. In addition, inhibition of p32 in Hela cells expressing mutant hCHCHD2 attenuated the mitochondrial fragmentation phenotype and abnormal mitochondrial function. In *Drosophila* and Hela cells expressing mutant hCHCHD2, the DRP-1 expression was upregulated and treatment with the p32-inhibitor was able to reduce the DRP-1 level. We further validated our data using human fibroblast derived neural precursor cells of *hCHCHD2-Arg145Gln*, which showed the effectiveness of p32-inhibitor in reducing mitochondrial fragmentation and restoring mitochondrial function as well as reducing α-synuclein level. Dopaminergic neurons derived from iPSCs harboring the *hCHCHD2-Arg145Gln* mutation showed reduced numbers of TH expressing neurons, shorter neurite extensions, mitochondrial fragmentation and lower ATP level, which were rescued by treatment with p32-inhibitor. Taken together, our study identified p32 as a modulator of CHCHD2-linked pathology in PD and inhibition of the p32 pathway may be a potential therapeutic approach for CHCHD2-linked PD as well as diseases involving mitochondrial dysfunction.

## Methods

### *Drosophila* husbandry and stocks

Standard food media containing yeast, cornmeal, dextrose, bacto agar and the anti-microbial agent were prepared. The p32-inhibitor containing food was prepared by mixing it into the prepared fly media together with a drop of food coloring to assure uniform mixing. Fly stocks were kept at 20 °C and crosses were performed at 25 °C in a humidified incubator. The *Drosophila* lines *yw* (control), *GMR-GAL4*, *Ddc-GAL4* and *24B-GAL4* were obtained from Bloomington *Drosophila* stock center (USA). The hCHCHD2 transgenic lines were generated previously [51]. The *p32-RNAi* line was from the Vienna *Drosophila* Resource Center.

### Drosophila brain fixation and immunohistochemistry

The adult *Drosophila* brains were dissected in PBS, fixed (4% formaldehyde), washed (PBT: PBS + 0.1% Triton X-100), blocked (3% BSA in PBT) for 1 h at room temperature/RT and then incubated in the primary antibody (rabbit anti-Tyrosine Hydroxylase/TH/Sigma, 1:1000 dilution) overnight at 4 °C. On the next day, samples were washed and incubated in the secondary antibody (Cy3-conjugated goat anti-rabbit/Jackson Immunoresearch, 1:500 dilution) for 1.5 h at RT, washed and added the Vectashield mounting media (Vector Laboratories). Stained samples were analyzed using Leica TCS SP8 inverted microscope. Images were collected as z-stacks, each with the size of 1 µm.

### *Drosophila* negative geotaxis and lifespan analyses

Locomotion/climbing ability was analyzed using negative geotaxis assay. Approximately 20–30 flies were left to acclimatize in the climbing tube and the percentage of flies that were able to climb over 20 cm height in a minute was recorded. At least three experimental repeats were performed for each group. For each repeat, climbing experiment was performed three times and the results were averaged. Climbing ability was performed at day 45 and day 55 of aging on the same groups of samples. At day 55, the numbers of experimental repeats might reduce due to loss of some flies with aging but a minimum of three experimental repeats were analyzed. For lifespan analysis, approximately 30 adult flies were put in each vial and they were transferred to fresh vials every 2–3 days. At each transfer, the numbers of dead flies were recorded. Lifespan analysis was followed until all the flies in each vial had died. These experiments were performed in a blinded manner, with samples labelled with codes such that the individuals performing the experiments would not be able to identify the genotypes and the treatment conditions.

### HPLC analysis

The dopamine level in the *Drosophila* head was measured using HPLC (Thermo Fisher Scientific, Ultimate 3000), according to manufacturer’s instructions. Briefly, 10 fly heads were homogenized in 2% perchloric acid, sonicated 3 to 5 times on ice (each time for about 20 s) and centrifuged at 10,000 rpm for 10 min at 4 °C. The lysates were filtered through the 1 µm filter tubes (MERK). 25 µl of the lysates were injected into the HPLC. The dopamine standard was used to identify the dopamine peak on the chromatograph and for quantitation purpose. The dopamine standard used was of 1 µM, 2.5 µM, 5 µM, 10 µM, 25 µM, 50 µM, 100 µM, 250 µM, 500 µM and 1000 µM concentration range.

### Western Blot and co-immunoprecipitation analysis

Approximately 30–50 adult *Drosophila* heads were homogenized in protease inhibitor (Roche) containing M-PER buffer (Thermo Fisher Scientific). Cell debris were removed by centrifugation and the amounts of total proteins were measured (BCA protein assay/Thermo Fisher Scientific). Equal amounts of total proteins were incubated in 2 × Laemmli sample buffer (Bio-Rad), denatured by heating to 95 °C for 5 min and loaded onto SDS –PAGE. Proteins were transferred to the nitrocellulose membrane using iBlot 2 Dry Blotting System (Thermo Fisher Scientific). The membrane was blocked (5% non-fat dry milk in 1 × PBST), incubated in the primary antibody overnight at 4 °C, washed and incubated with the secondary antibody for 1 h at RT. Protein bands were visualised by chemiluminescence substrate (SuperSignal West Femto Maximum Sensitivity HRP Substrate kit/Thermo Fisher Scientific). For Western Blot experiments involving cells extracts, the cells were lysed using RIPA buffer (Thermo Fisher Scientific) and processed similarly as described above. For all samples, 30 µg of total proteins were loaded on each gel lane. Protein bands were quantitated using the Image J software. The net values of each protein bands were normalised against the net values of the corresponding loading controls.

Co-immunoprecipitation (Co-IP) assays were performed according to the manufacturer’s instruction. Briefly, the EZview Red Anti-c-Myc Affinity Gel bead slurries (Sigma) were equilibrated by washing with the ice-cold RIPA lysis buffer. Approximately 60 *Drosophila* heads or 3 × 10^6^ Hela cells were homogenized in the lysis buffer containing protease inhibitor (Roche) and centrifuged at 13,500 rpm for 20 min (4 °C). The supernatants were mixed to the equilibrated beads then left to rotate for 2 h (4 °C) and centrifuged at 8,200 × *g* for 30 s. The bead pellets were washed and the protein complexes were eluted then denatured at 95 °C for 5 min and subjected to Western blot analysis.

Primary antibodies used: rabbit anti-CHCHD2 (Proteintech, 1:1000 dilution), mouse anti-P32/GC1qR (Abcam, 1:1000 dilution), mouse anti-MYC (Cell Signalling, 1:1000 dilution), rabbit anti-HA (Cell Signalling, 1:1000 dilution), rabbit anti-Tyrosine Hydroxylase (Sigma, 1:1000), mouse anti-Tubulin (Sigma, 1:10,000 dilution), rabbit anti-DRP1 (Cell Signalling, 1:1000 dilution), mouse anti-OPA1(BD Biosciences, 1:1000 dilution), rabbit anti-α-synuclein (Abcam, 1:1000 dilution), mouse anti-HSP90 (Origene, 1:1000 dilution) and mouse anti-Actin (Sigma, 1:10,000 dilution). Secondary antibodies used: HRP-conjugated goat anti-mouse IgG (Santa Cruz Biotechnology, Inc., 1:5000 dilution) and anti-rabbit IgG (Santa Cruz Biotechnology, Inc., 1:5000 dilution).

### RT-qPCR

Total RNA was extracted from 30 fly heads using the TRIzol reagent (Life Tech), purified with DNAse (Ambion) and the concentration measured using the NanoDrop One (Thermo Fisher Scientific). RNAs were reversed transcribed using a High-Capacity Reverse Transcription kit (Applied Biosystems). qPCR was performed using GoTaq® qPCR Master Mix (Promega) and the 7500 Fast Real-Time System (Applied Biosystems). qPCR primer sequences were *dDrp1*: forward 5’-GAGTGGAACGCGACCAAAG-3’ and reverse 5’-AGGATCGTCCCACAACGCT-3’, *dOpa1*: forward 5’-TCCCCAGATTGCGCGAG-3’ and reverse 5’-CAGCGGGCAGATAGATGCTT-3’, *rp-49*: forward 5’-GACGCACTCTGTTGTCGATAC-3’ and reverse 5’-TACAGGCCCAAGATCGTGAAG-3’. Relative gene expression was calculated using the 2(-Delta Delta C(T)) method [56].

### Immunostainings of cells and imaging

Cells were seeded on 18 mm round coverslips in the 12-well plates, grown for 72 h in the absence or presence of p32-I, incubated with fixation buffer (Biolegend) for 20 min at RT, permeabilized and blocked for 45 min. Fixed cells were incubated in primary antibodies for 3 h at RT, washed and incubated with secondary antibodies for 1 h. Samples were washed and incubated with DAPI (Invitrogen) for 20 min at RT in the dark. Coverslips containing stained samples were mounted onto glass slides using FluorSave ™ reagent (Calbiochem). Primary antibodies used: rabbit anti-TOM20 (Santa Cruz, 1:1000 dilution), mouse anti-MYC (Santa Cruz, 1:250 dilution). Rabbit anti-Nestin (Proteintech, 1:50 dilution), rabbit anti-Sox2 (Millipore, 1:100 dilution). Secondary antibodies used: Alexa Fluor 488 and Alexa Fluor 594 (Invitrogen). Samples were analyzed using Leica TCS SP8 Inverted Microscope.

### Culture and generation of stable Hela cell lines and p32 suppression by siRNA

Hela cells were cultured in growth medium comprising of Dulbecco’s modified Eagle medium (Gibco-Thermo Fisher Scientific), 10% fetal bovine serum (Biowest), 1% MEM amino acid solution, 1% sodium pyruvate and 1% penicillin/streptomycin (Thermo Fisher Scientific), in the T75 flask at 37 °C incubator with 5% CO_2_ atmosphere. Cells were harvested with 0.05% Trypsin–EDTA and washed with 1 × PBS. Hela cells which stably expressed hCHCHD2 were generated by transfection with the hCHCHD2-MYC plasmid DNA followed by selection in G418 (Thermo Fisher Scientific). Single cell colonies were picked and proliferated. Cells from colonies that expressed moderate and comparable level of hCHCHD2 were used. For p32 suppression, scrambled siRNA (ON-TARGET plus Non-targeting Pool, D-001810–10-05, Dharmacon) and siRNA targeting p32 (SI04164692, QIAGEN) were transfected into Hela cells expressing wild type or mutant hCHCHD2 using Lipofectamine RNAiMAX Transfection Reagent (Thermo Fisher Scientific) for 72 h in the growth medium before processing for Western Blotting.

### Generation and culture of neural precursor cell lines, dopaminergic neurons and p32-I treatment

The *hCHCHD2-Arg145Gln* homozygous KI hiPSC and its isogenic control were derived from human fibroblast line and differentiated into neural precursor cells/NPCs and then mature dopaminergic neurons (iXCells Biotechnologist). Presence of *hCHCHD2-Arg145Gln* was verified through sequencing using the following *CHCHD2* primers: *F*orward 5’-AATGTTTTCACTTCCCATGTTAATAGTTG-3’ and Reverse 5’-AATGACTAGAAACCTCCGGCCC-3’. Culturing of the NPCs and the dopaminergic neurons was done according to manufacturer’s instructions. Briefly, cells were seeded onto Poly-L-Ornithine /Laminin (Sigma) coated plate containing Human NSC growth medium or DA maturation medium (iXCells Biotechnologist) with 10uM Y-27632 (Miltenyi Biotec). The NPCs were grown to ~ 80% confluency (at 37 °C, 5% CO_2_) and cells from early passages were used. The dopaminergic neurons were incubated in the DA maturation medium for 48 h and then incubated with 1 µM of p32-I. Both cultures were grown in the presence of p32-I for 3 days, washed with 1 × PBS and dissociated using Accutase (Thermo Fisher Scientific).

### p32 inhibitor generation and testing

The p32 protein was identified to be the receptor for LyP-1 (a tumor homing peptide) [57]. Thus, we custom-generated an inhibitor that binds to the LyP-1 binding domain of p32 (also called p32-inhibitor/p32-I) which is of high purity (Enamine Ltd.). For cell culture experiments, the optimal dose of p32-I which were devoid of cytotoxicity were tested on Hela cells using a range of p32-I concentrations (0 µM, 1 µM, 2.5 µM, 5 µM, 7.5 µM, 10 µM, 25 µM, 50 µM, 75 µM, 100 µM and 250 µM in DMSO). Cell viability was quantified using CellTiter-Glo Luminescent Cell Viability Assay (Promega) and the luminescence levels were recorded using PerkinElmer Multimode plate reader. The effectiveness of the chosen concentration of p32-I (1 µM) was verified by its ability to reduce p32 protein levels. For the *Drosophila* experiments, 25 µM and 50 µM of p32-I were initially tested in small scale experiments for their ability to show phenotypic rescue in the transgenic *Drosophila* expressing mutant hCHCHD2. The p32-I concentration of 25 µM was found to be effective for phenotypic rescue and posed less toxicity. It was mixed in the food at the final concentration of 25 µM and fed to the adult *Drosophila*.

### Transmission electron microscopy (TEM) analysis

TEM analyses were performed using the indirect flight muscles of the adult flies. To do this, thoraces of 45-day-old flies were hemisected and fixed with a solution containing 2.5% glutaraldehyde in 0.1 M phosphate buffer (pH: 7.2, with 0.1% Tween-20). Samples were mailed to the Electron Microscopy Research Services at Newcastle University (Newcastle upon Tyne, UK) for further processing, sectioning and imaging [58]. Briefly, samples were further processed by post-fixing in 1% osmium tetroxide, washed, followed by dehydration in a graded series of acetone. Samples were then embedded in 100% fresh resin and left to polymerize at 60 °C, then sectioned. The ultrathin sections were stained with uranyl acetate and lead citrate for better image contrast. Samples were then viewed under transmission electron microscope. Images from three experimental repeats were analyzed.

### ATP measurement

ATP level was measured using CellTiter-Fluor Cell Viability Assay which was multiplexed with CellTiter-Glo Luminescent Assay, according to manufacturer’s instructions. Briefly, approximately 3000 cells were dispensed into each well of a white 96-well plate in the absence or presence of p32-I, grown for 72 h in the 37 °C incubator under 5% CO_2_ then added 20 µl of CellTiter-Fluor reagent (Promega). The plate was incubated for another hour and the fluorescent intensity was measured (390 nm-_Ex_/505 nm-_Em_). Then 100 µl of CellTiter-Glo (Promega) reagent was added to the cells and incubated for 10 min at RT. Both the fluorescence and the luminescence levels were measured with the EnSpire multimode microplate reader (PerkinElmer). The ATP level was normalized to the corresponding cell numbers of the same group.

### H_2_O_2_ assay

H_2_O_2_ assay was performed using the Amplex™ Red Reagent (Thermo Fisher Scientific) according to manufacturer’s instructions. Briefly, *Drosophila* head extracts were homogenized in sodium phosphate buffer (pH 7.4), centrifuged at 13,000 rpm for 15 min (4 °C) and the supernatant was added into a 96 well, black flat- bottomed plate. For the calibration standards, H_2_O_2_ concentration used were 0 µM, 0.0625 µM, 0.125 µM, 0.25 µM, 0.5 µM, 1 µM, 2 µM, 4 µM, 8 µM. Blanks were samples without H_2_O_2_. 50 µl of Amplex red (100 µmol) and HRP (0.2 U/ml, Invitrogen) were added to the plates containing standards, controls and samples. The plates were then incubated for 30 min at RT and protected from light. The fluorescence outputs were measured using the EnSpire Multimode Plate Reader at Ex/Em of 530/590 nm. All readings were corrected against the mean background fluorescence.

### Morphological analysis

To quantitate the percentage of fragmented mitochondria, cells were first grown on coverslips, fixed and immunostained with the anti-TOM20 antibody and observed under confocal microscope. In both the Hela cells and the isogenic cell lines, fragmented mitochondria appeared as punctate foci, and which could be easily differentiated from non-fragmented mitochondria (circles/before fusion or elongated/after fusion). Non-overlapping sections of each coverslip of cells were randomly picked for analysis. The percentage of mitochondrial fragmentation was calculated by dividing the number of cells with fragmented mitochondria with the total number of cells scored then multiplied by 100. For each group, quantitation was derived from three experimental repeats and for each repeat, at least 100 mitochondria were analyzed. For quantitation of neurite length of the dopaminergic neurons, the images were first changed to 8-bit and phase contrasted. The individual neurite length was semi-automatedly traced and quantitated using the Fiji/ImageJ plug-in (NeuronJ toolset). A total of 60 neurons from each treatment group were quantitated. For quantitation of mitochondrial branch length, the images were first changed to 8-bit and threshold was set before measurement. The length of the mitochondria was quantitated using the Fiji/ ImageJ plug-in (Mitochondrial Network Analysis/MiNA toolset). A total of 60 HeLa cells and 60 NPCs per condition were quantitated. For each condition, there were three experimental repeats. Statistical significance was determined using One-Way ANOVA with Bonferroni post-hoc test.

### Statistical analysis

All data are presented as mean ± standard error of the mean (SEM). Negative geotaxis assay was analysed using Pearson Chi-square (dƒ = 1). Survival assay was analysed using Kaplan–Meier with Cox regression (95% confidence interval). Differences between two groups were analysed using two-tailed Paired Samples *t*-test. For comparison of multiple groups, One-Way ANOVA with Bonferroni post-test was used. The statistics, numbers of experimental repeats performed and numbers of samples analysed are stated in the figure legends. Statistical analyses were performed using IBM-SPSS Statistical 21 software. Statistically significant differences were defined as ** for p < 0.01 (highly significant) and * for p < 0.05 (significant). For all experiments, data were obtained from at least three biological repeats and each biological repeat was accompanied by three technical repeats.

## Results

### p32 knockdown mitigates and p32 overexpression exacerbates the phenotypes of mutant hCHCHD2 transgenic *Drosophila*

**Fig. 1:**
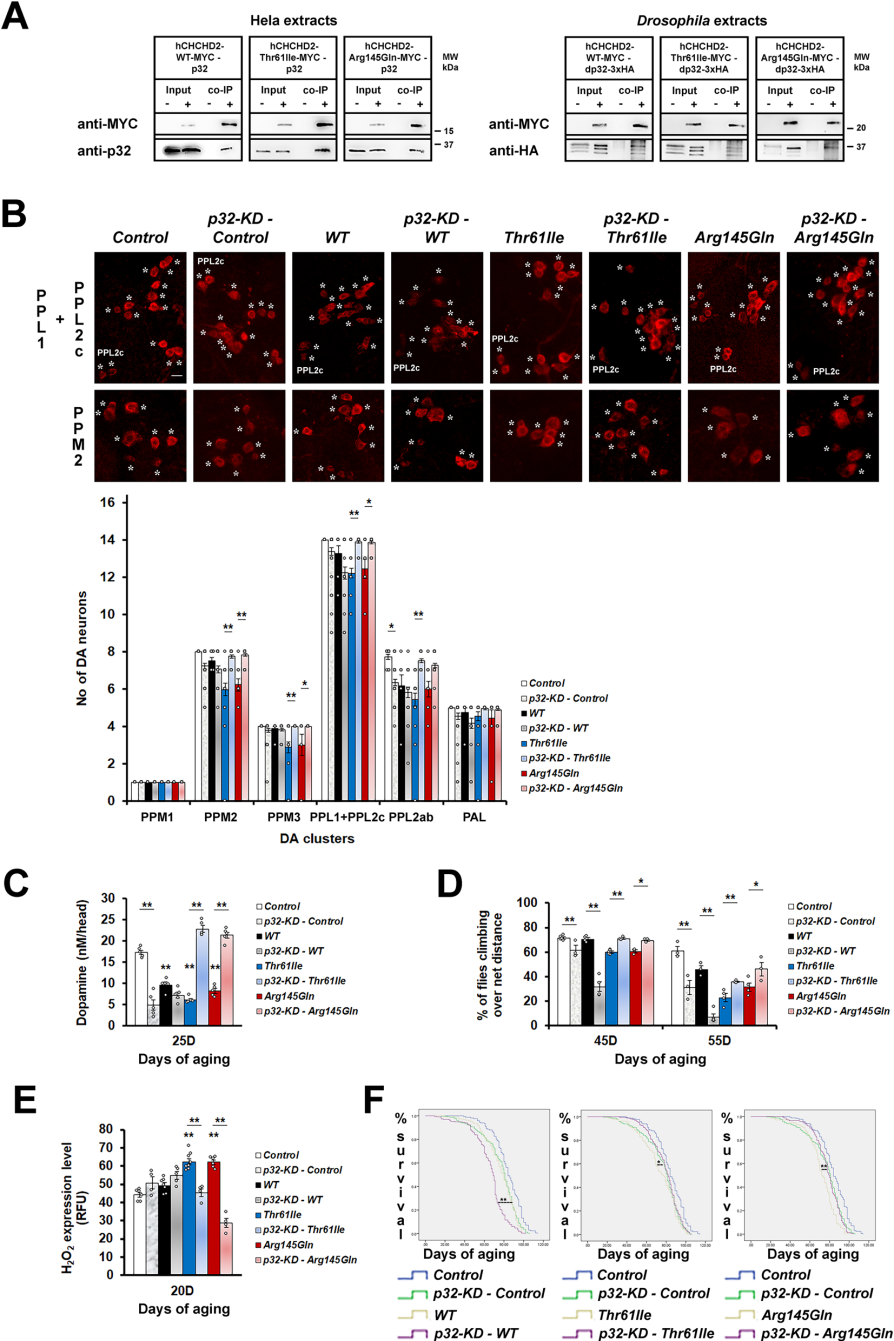
p32-knockdown ameliorates the phenotypes of mutant hCHCHD2 transgenic *Drosophila*. **A** Co-immunoprecipitation of hCHCHD2 and p32 (n = 3). Negative controls: Hela cells expressing MYC; *Drosophila* expressing dp32-3xHA (*GMR-GAL4*). The multiple bands present in the anti-HA immunoblots are due to the 3xHA tag of the expressed p32-protein. **B** Representative confocal images of 40-day-old adult *Drosophila* brains stained for the dopaminergic neuronal marker, TH (tyrosine hydroxylase, *) in the PPL1 + PPL2c and PPM2 clusters, and quantitation [59]. Due to their close proximity, the twelve PPL1 and two PPL2c neurons are grouped together in the quantitation (14 neurons total). For the PPM3 cluster, only the four strong TH-positive neurons are quantitated. For each group, a total of 9 to 38 brain samples were analyzed, with more samples analyzed for the group with p32-KD (n = 3; One-way ANOVA with Bonferroni post hoc test). These experiments were done simultaneously with those in Fig. 2 and hence, both share the same PPL1 + PPL2c images of *Control*, *WT*, *Thr61Ile* and *Arg145Gln*. Scale bar = 10 µm. **C** Dopamine levels as measured by HPLC. A total of 50 head samples for each group were analyzed (n = 5; One-way ANOVA with Bonferroni post hoc test). **D** Climbing ability was monitored at day 45 and 55, using negative geotaxis assays. For each group, approximately 90 to 150 flies were examined at day 45 of aging (n = 3 for p32-KD – Control, p32-KD – Thr61Ile, p32-KD – Arg145Gln groups; n = 4 for WT, p32-KD – WT, Arg145Gln groups; n = 5 for Control, Thr61Ile groups). Statistics used is Pearson Chi-Square (df = 1). **E** Measurement of H_2_O_2_ levels (n = 4–8, One-way ANOVA with Bonferroni post hoc test). **F** Survival curves. For each group, a total of 159 to 347 flies were scored (n = 5, Kaplan–Meier with Cox Regression, 95% Confidence Interval). In **(B-F)**. Control is *Ddc-Gal4/* + . n = experimental repeats. Data are presented as mean ± SEM, ^*^p < 0.05; ^**^p < 0.01

From co-immunoprecipitation (co-IP) experiments, we found that Hela cells expressing wild type or mutant hCHCHD2 (Thr61Ile or Arg145Gln) were able to bind to p32 (Fig. 1A). By expressing hCHCHD2-MYC and dp32-HA in the *Drosophila* eye with the *GMR-GAL4*, we demonstrated in vivo a conserved and direct physical interaction between the human CHCHD2 and the *Drosophila* p32. In accordance with the in vitro result, mutant hCHCHD2 retained the ability to bind to dp32 in vivo (Fig. 1A).

To examine the functional consequences of CHCHD2 and p32 interaction, we modulated the expression levels of p32 through knockdown of p32 by RNA interference (RNAi) or overexpression of p32 in *Drosophila* which also expressed the different hCHCHD2 variants. Previously, we showed that flies expressing *hCHCHD2*-*Thr61Ile* or *hCHCHD2*-*Arg145Gln* in the dopaminergic and the serotonergic neurons exhibited shortened lifespan, locomotor impairment and degeneration of dopaminergic neurons [51]. We quantitated the main clusters of dopaminergic neurons (PPM1, PPM2, PPM3, PPL1 + PPL2c, PPL2ab and PAL [59]) in the adult *Drosophila* brain which had been stained with the anti-tyrosine hydroxylase antibody. We found that reducing p32 level by overexpressing one copy of *p32-RNAi* in the control flies caused mild abnormalities. However, flies that concurrently expressed mutant *hCHCHD2* and *p32-RNAi* had normal numbers of dopaminergic neurons (Fig. 1B). By performing high performance liquid chromatography (HPLC), we found that hCHCHD2 transgenic flies had much reduced dopamine levels as compared to the control (Fig. 1C). Knockdown of p32 increased the dopamine levels of the mutant but not of the wildtype hCHCHD2 transgenic flies (Fig. 1C). p32 knockdown also improved the locomotor function (Fig. 1D), reduced the oxidative stress (Fig. 1E) as well as lowered the mortality rate and extended the lifespan of mutant hCHCHD2 transgenic flies (Fig. 1F). Surprisingly, p32 knockdown did not rescue and instead worsened the previously reported old age-associated phenotypes of wild type hCHCHD2 transgenic flies such as the climbing ability and the lifespan (Fig. 1D and F) [51].

**Fig. 2:**
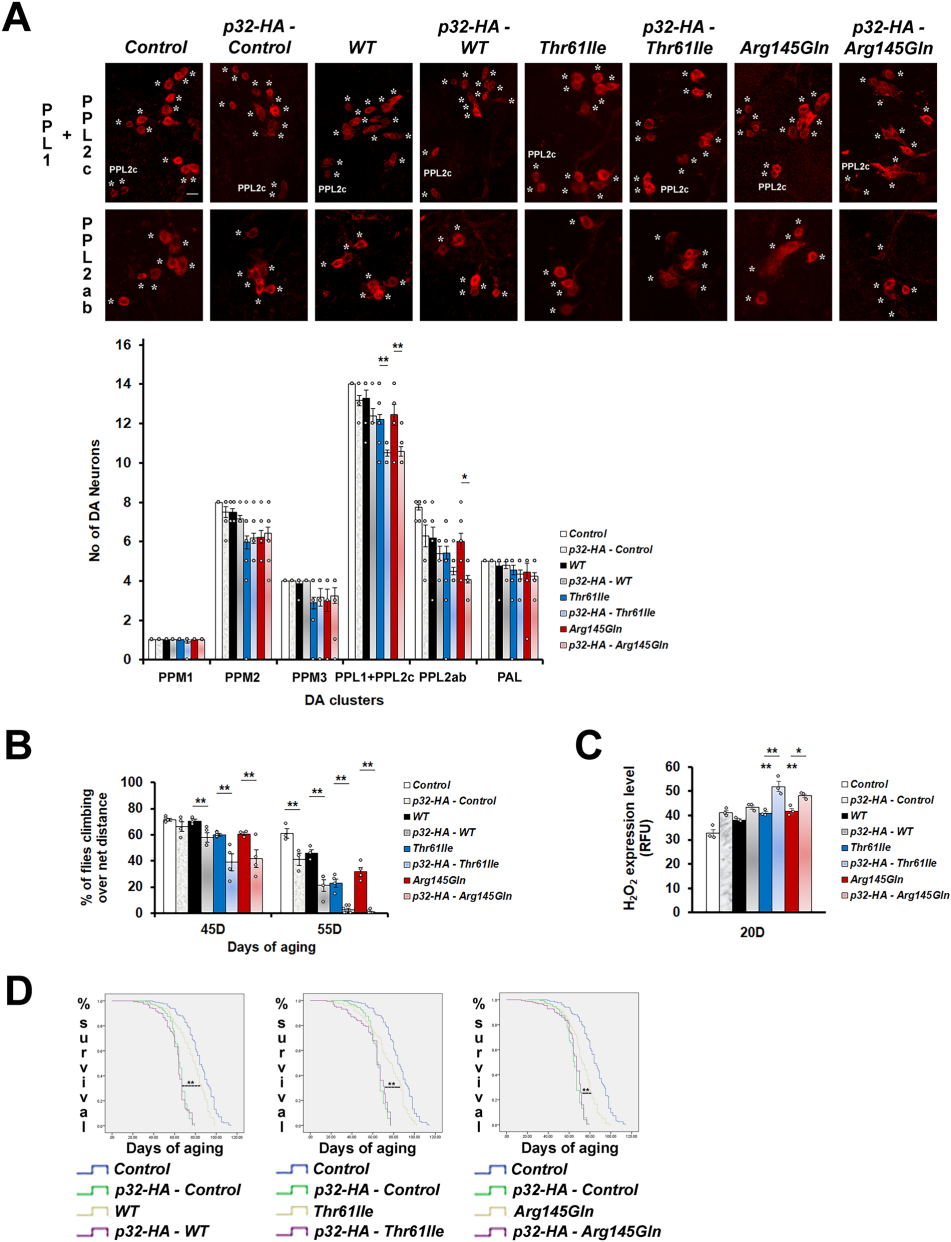
p32 overexpression exacerbates the phenotypes of hCHCHD2 transgenic *Drosophila*. **A** Representative confocal images of the 40-day-old adult *Drosophila* brains stained for TH expression (*), in the PPL1 + PPL2c and PPL2ab clusters, and quantitation. For each group, a total of 6 to 24 brain samples were analyzed (n = 3, One-way ANOVA with Bonferroni post hoc test). These experiments were done simultaneously with the experiments in Fig. 1 and hence, both share the same PPL1 + PPL2c images of *Control*, *WT*, *Thr61Ile* and *Arg145Gln*. Scale bar = 10 µm. **B** Climbing ability was monitored in adult *Drosophila* aged to 45 days and 55 days, using negative geotaxis assay. For each group, approximately 90 to 150 flies were examined at day 45 of aging (n = 3 for p32-HA – WT group; n = 4 for p32-HA – Control, WT, Arg145Gln, p32-HA –Arg145Gln groups; n = 5 for Control, Thr61Ile, p32-HA – Thr61Ile, groups). Statistics used is Pearson Chi-Square (df = 1). **C** Measurement of H_2_O_2_ levels (n = 3, One-way ANOVA with Bonferroni post hoc test). **D** Survival curves. For each group, the total numbers of flies scored are between 130 and 218 (n = 5, Kaplan–Meier with Cox Regression, 95% Confidence Interval). In all panels, *Ddc-GAL4* was used to drive the expression of hCHCHD2-MYC and dp32-HA. Control line is of genotypes *Ddc-Gal4/* + . n = experimental repeats. Data are presented as mean ± SEM, ^*^p < 0.05; ^**^p < 0.01

Since knockdown of p32 rescued the phenotypes of mutant hCHCHD2 transgenic *Drosophila*, we postulated that overexpression of p32 would cause the opposite effects. As expected, overexpression of p32 exacerbated the DA neuron degeneration (Fig. 2A), the climbing impairment (Fig. 2B), the oxidative stress (Fig. 2C), as well as the mortality rates (Fig. 2D) associated with flies expressing hCHCHD2.

### Knockdown of p32 differentially controls the expression of wild type and mutant hCHCHD2

**Fig. 3:**
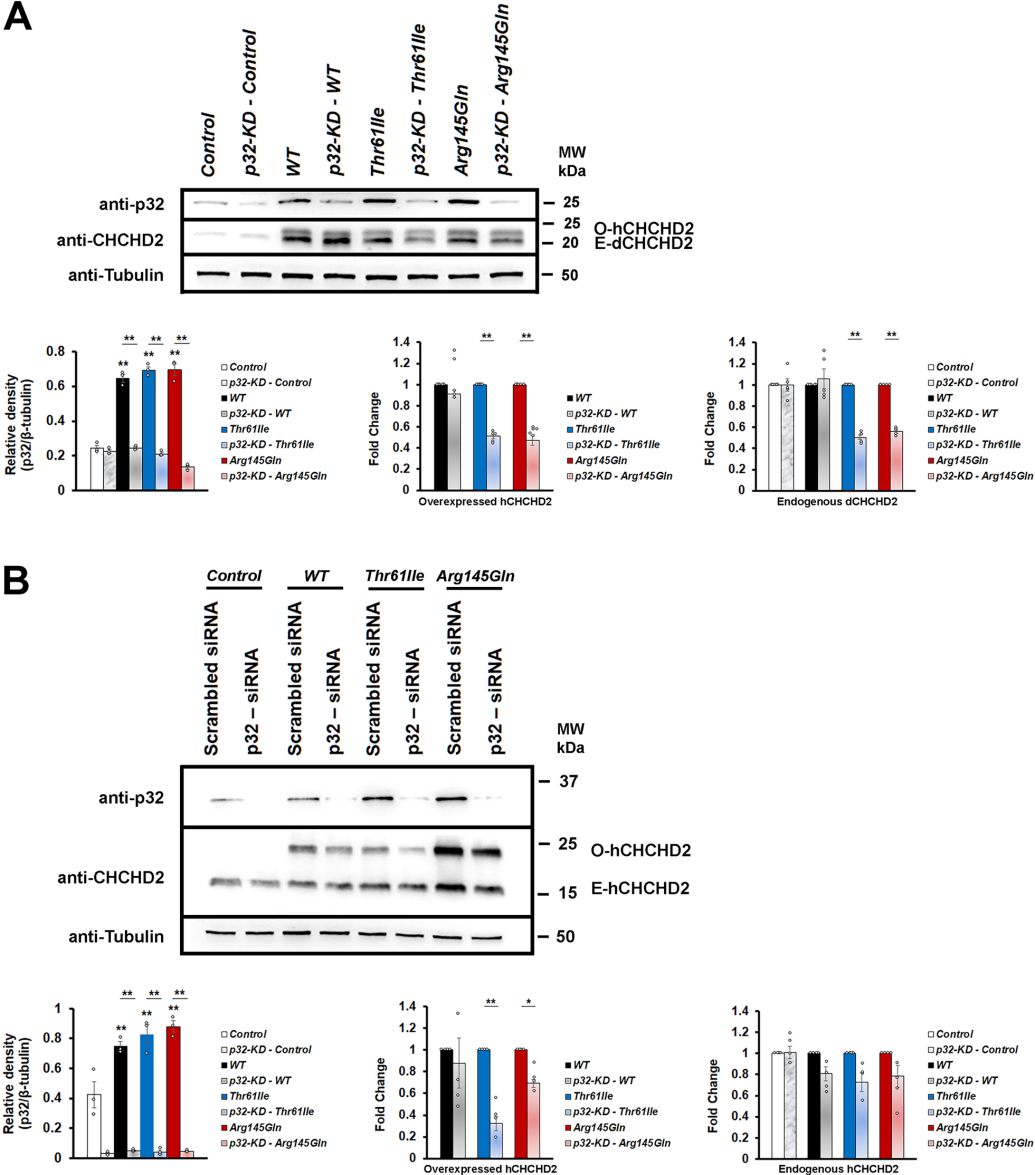
p32-knockdown reduces mutant CHCHD2 protein levels. **A** Representative Western blots of p32 and CHCHD2 expressions in *Drosophila*. Expression of wild type or mutant hCHCHD2 in *Drosophila* results in increased endogenous p32 level. Densitometric analysis of p32 expressions (shown as relative density, n = 3; One-way ANOVA with Bonferroni post hoc test) and CHCHD2 expressions (shown as fold change, n = 5; two-tailed Paired Samples *t*-test). Both the overexpressed human CHCHD2 (O-hCHCHD2) and the endogenous *Drosophila* CHCHD2 (E-dCHCHD2) expressions are shown. Negative control is of genotype *Ddc-Gal4/* + . **B** Representative Western blots showing p32 and CHCHD2 expressions in the Hela cells. As in *Drosophila*, expression of wild type or mutant hCHCHD2 in Hela cells results in increased endogenous p32 level. Densitometric analysis of p32 (shown as relative density, n = 3; One-way ANOVA with Bonferroni post hoc test) and CHCHD2 expressions (shown as fold change, n = 4; two-tailed Paired Samples *t*-test). Both the over-expressed human CHCHD2 (O-hCHCHD2) and the endogenous human CHCHD2 (E-hCHCHD2) expressions are shown. Negative control is Hela cells expressing MYC alone. In both (**A**, **B**), the molecular weight markers are indicated in kDa. The p32 and the CHCHD2 expressions are detected using the anti-p32 and the anti-CHCHD2 antibodies. β-tubulin is used as the loading control. n = experimental repeats. Data are presented as mean ± SEM. ^*^p < 0.05; ^**^p < 0.01

Physical interaction between CHCHD2 and p32 suggests that they may control the expression of each other. By performing Western blotting, the p32 protein levels were found to be increased in the transgenic hCHCHD2 *Drosophila* and which could be lowered by p32-knockdown (Fig, 3A). In all the transgenic *Drosophila*, expression of hCHCHD2 were higher than the endogenous dCHCHD2 expression in the controls. The increased endogenous dCHCHD2 expression in each transgenic line was likely due to a known role of CHCHD2 to autoregulate itself [60]. Hence, the results also suggest that the hCHCHD2 mutations being analyzed did not affect their autoregulatory ability. We found that although p32-knockdown did not have significant effects on the level of wildtype CHCHD2, it could significantly reduce the levels of both the overexpressed mutant hCHCHD2 and the corresponding endogenous dCHCHD2 (Fig. 3A).

To support our in vivo observations in *Drosophila*, we extended our protein expression analysis using the human Hela cell lines. The p32-siRNAi was employed to knock down p32 in cultured cells which also expressed wild type or mutant hCHCHD2-Myc. The cells expressing Myc alone was used as the control cells. Overexpression of hCHCHD2 in the Hela cell lines similarly increased the p32 levels and which could be effectively reduced by the p32-siRNA (Fig. 3B). Similar to the observation in *Drosophila*, the expression level of hCHCHD2-Thr61Ile appeared to be lower as compared to those of hCHCHD2-WT and hCHCHD2-Arg145Gln. p32-knockdown was able to significantly reduce the levels of overexpressed hCHCHD2-Thr61Ile and hCHCHD2-Arg145Gln but not of the hCHCHD2-WT (Fig. 3B). Although statistically insignificant, p32-knockdown reduced the levels of endogenous hCHCHD2.

### p32-inhibitor (p32-I) suppresses the phenotypes of mutant hCHCHD2 transgenic *Drosophila*

**Fig. 4:**
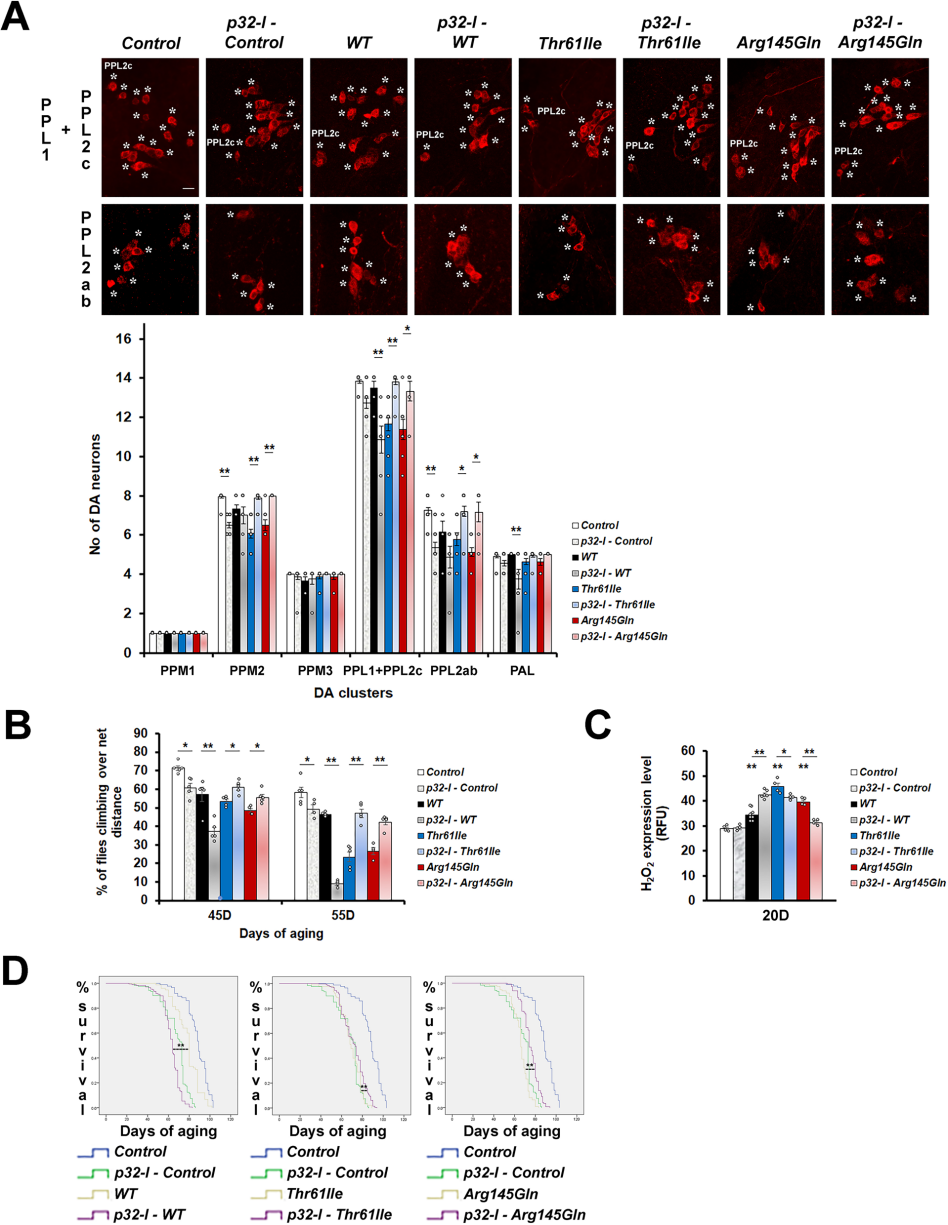
p32-inhibitor treatment ameliorates the phenotypes of mutant hCHCHD2 transgenic *Drosophila*. **A** Representative confocal images of the 40-day-old adult *Drosophila* brains stained for TH expression (*) in the PPL1 + PPL2c and PPL2ab clusters, and quantitation. For each group, a total of 6 to 20 brain samples were analyzed (n = 3, One-way ANOVA with Bonferroni post hoc test). **B** Climbing ability was monitored in adult *Drosophila* aged to 45 days and 55 days using negative geotaxis assay. For each group, approximately 120 to 150 flies were examined at day 45 of aging (n = 5 for all groups, except n = 4 for Arg145Gln group). Each experimental repeat was accompanied by three technical repeats. Statistics used is Pearson Chi-Square (df = 1). **C** Measurement of H_2_O_2_ levels in the transgenic *Drosophila* expressing hCHCHD2, with or without p32-I treatment (n = 4 for all groups, except n = 6 for p32-I – WT group and n = 8 for WT group). Statistics used is One-way ANOVA with Bonferroni post hoc test. **D** Kaplan–Meier analysis of survival rates in the transgenic *Drosophila* expressing hCHCHD2, with or without p32-I treatment. For each group, the total numbers of flies scored are between 158 and 276 (n = 3). The concentration of p32-I used is 25 µM. n = experimental repeats. Data are presented as mean ± SEM. ^*^p < 0.05; ^**^p < 0.01

To investigate the ability of p32-inhibition in ameliorating the phenotypes of our *hCHCHD2* disease models, we custom-generated a p32-inhibitor (p32-I). The p32-I was first tested on cells and the 1 µM concentration was found to be non-toxic (Fig. S1A) and was effective in lowering the p32 protein level (Fig. S1B). For treatment in *Drosophila*, a higher concentration of p32-I (25 µM) was found to be effective. To determine if treatment with p32-I could mimic the knockdown effects of p32, we fed the *hCHCHD2* transgenic *Drosophila* with this inhibitor and found that it could restore the dopaminergic neuronal numbers in all affected clusters in the *Drosophila* expressing mutant hCHCHD2 (Fig. 4A). The p32-I treatment also improved the climbing ability (Fig. 4B), reduced the oxidative stress (Fig. 4C) and decreased the mortality rate and extended the lifespan (Fig. 4D) of transgenic mutant *hCHCHD2 Drosophila*. Similar to the knockdown effects, treatment of wildtype-hCHCHD2 transgenic *Drosophila* with p32-I resulted in worsening of all the phenotypes investigated.

**Fig. 5:**
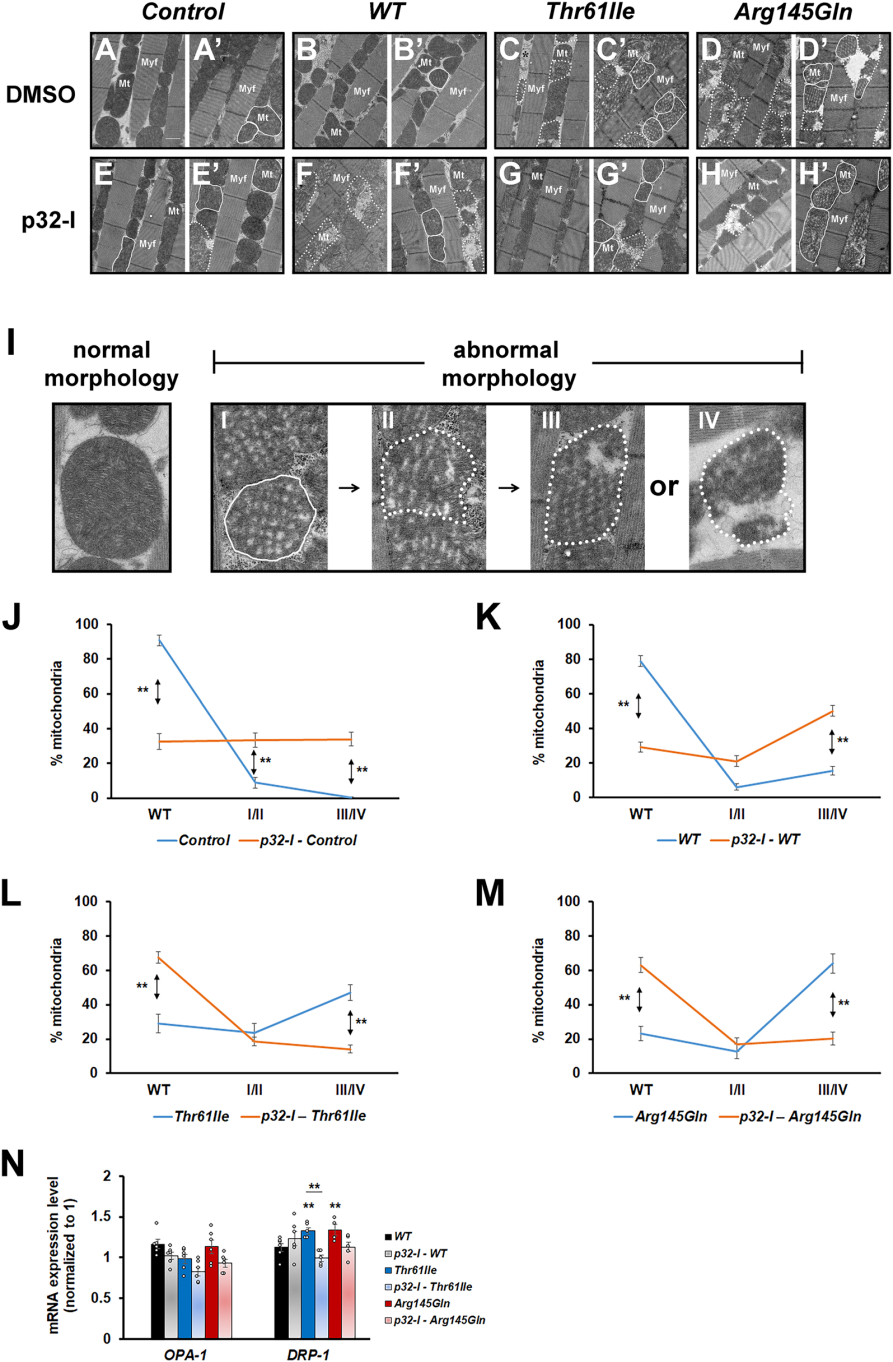
p32-inhibitor treatment ameliorates the muscle mitochondria morphological defects of mutant hCHCHD2 expressing *Drosophila*. **A**–**H’** Representative Transmission Electron Microscopy (TEM) images of the indirect flight muscles of the 45-day-old *Drosophila* expressing the indicated transgenes and which have been fed with either DMSO or 25 µM of p32-I. Control is of genotype *24B-GAL4/* + . Two images for each category are shown for clarity. Myf: myofibril, Mt: mitochondria. Solid lines encircle mitochondria with spongy appearance and dotted lines encircle fragmented mitochondria. **I** The different categories of mitochondria are shown. Normal mitochondria appear densely packed and tubular while abnormal mitochondria appear less densely packed with spongy appearance (I, II) and fragmented (III, IV). **J**–**M** Quantification of the mitochondrial morphology. Graph plots showing the percentages of the different categories of mitochondria (normal/WT, I/II and III/IV). For each group, a total of 293 to 969 mitochondria were scored (n = 3). Statistics used is two-tailed Paired Samples *t*-test. **N** qRT-PCR analysis of *OPA-1* (n = 6 for all groups) and *DRP-1* expressions (n = 4 for Arg145Gln group; n = 5 for Thr61Ile, p32-I – Thr61Ile, p32-I – Arg145Gln groups; n = 6 for WT, p32-I – WT groups). Statistics used is One-way ANOVA with Bonferroni post hoc test. n = experimental repeats. Data are presented as mean ± SEM. ^*^p < 0.05; ^**^p < 0.01

Pharmacological inhibition of p32 also improved the muscle mitochondrial morphology of the mutant hCHCHD2 transgenic *Drosophila*, as shown by reduced mitochondrial fragmentation (Fig. 5C/C’, 5G/G’ for *hCHCHD2-Thr61Ile* and 5D/D’, 5H/H’ for *hCHCHD2-Arg145Gln*). By categorizing the mitochondrial morphology into normal (WT), abnormal (with spongy appearance, type I/II) and fragmented (type III/IV), we found that there was a shift from the presence of more mitochondria with fragmentation towards more mitochondria with normal morphology in the mutant *hCHCHD2* transgenic *Drosophila* fed with the p32-I (Fig. 5L and M). The opposite shift from presence of more mitochondria with normal morphology towards more fragmented mitochondria could be seen in wild type *hCHCHD2* transgenic flies fed with the p32-I (Fig. 5K). We also investigated the effects of p32-inhibition on mitochondrial dynamics by analyzing the expression of two key important genes, *OPA-1* (in fusion) and *DRP-1* (in fission). As effective antibodies against these two *Drosophila* proteins were not available, we monitored their mRNA levels. We found that *OPA-1* levels were not significantly altered in all the transgenic hCHCHD2 flies (Fig. 5N), suggesting that the fusion process was not affected. While the *DRP-1* level was not significantly affected in transgenic *Drosophila* expressing wildtype hCHCHD2, it was significantly elevated in the transgenic *Drosophila* expressing mutant hCHCHD2. The increased *DRP-1* level could be lowered to near normal level when these flies were fed with the p32-I, although statistical tests only showed significance for the hCHCHD2-Thr61Ile but not for the hCHCHD2-Arg145Gln transgenic flies (Fig. 5N). High levels of *DRP-1* mRNA expression had been linked to mitochondrial fragmentation [44].

### p32-I improves the mitochondrial morphology and function of Hela cells expressing mutant hCHCHD2

**Fig. 6:**
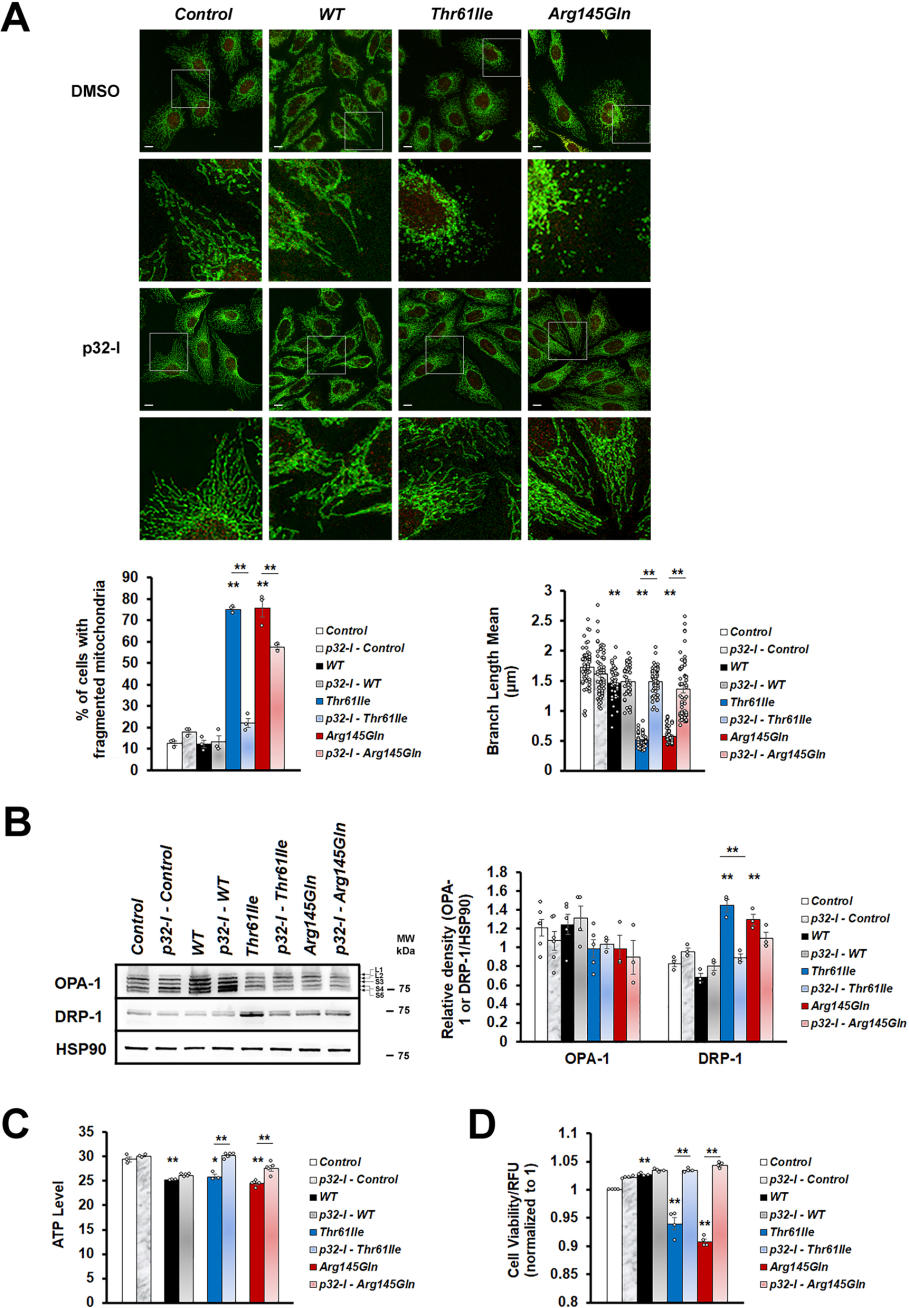
p32-inhibitor rescues mitochondrial morphological defects and functions of Hela cells expressing mutant hCHCHD2. **A** Representative confocal images of immunoreactivity for the mitochondrial marker (TOM20, green) and the MYC marker (red) in Hela cells. Magnified regions are boxed. Scale bar = 10 µm. Quantification of the mitochondrial morphology (n = 3). Measurement of mitochondrial branch length. **B** Representative Western blot and the quantitation of OPA-1 (n = 3 for p32-I – Thr61Ile, Arg145Gln, p32-I – Arg145Gln groups; n = 4 for p32-I – WT; n = 5 for WT, Thr61Ile Groups; n = 6 for Control, p32-I – Control groups) and DRP-1 (n = 3). The multiple bands of OPA-1 are the result of alternative splicing, which generates the long OPA-1 isoforms (L1 and L2) and the short OPA-1 isoforms (S3, S4 and S5). Molecular weight markers are indicated in kDa. **C** Representative graph showing ATP levels in the cells expressing hCHCHD2, with or without p32-inhibitor treatment (n = 3 for Control, p32-I – Control, WT, Thr61Ile groups; n = 5 for p32-I – WT, p32-I – Thr61Ile, Arg145Gln, p32-I – Arg145Gln groups). **D** Cell viability of Hela cells expressing hCHCHD2, with or without p32-inhibitor treatment (normalized to the control cells, n = 4). n = experimental repeats. Data are presented as mean ± SEM. Statistics used is One-way ANOVA followed by Bonferroni post hoc test. ^*^p < 0.05; ^**^p < 0.01

To corroborate the effects of p32-inhibition in our *Drosophila* hCHCHD2 models, we validated some of our findings using the Hela cell lines. Using TOM20 as the mitochondrial marker, we found that Hela cells expressing mutant hCHCHD2 showed fragmentation of the mitochondrial network into punctate cytoplasmic dots and which could be rescued by the p32-I treatment (Fig. 6A). Mitochondrial branches appeared much shorter in Hela cells expressing mutant hCHCHD2 and which was significantly improved after treatment with the p32 inhibitor. From the Western Blot experiments, we found that although OPA-1 levels were slightly lower in the cells expressing mutant hCHCHD2, they were not statistically significant when compared to the control (Fig. 6B). Hela cells expressing wildtype CHCHD2 showed higher level of OPA-1 and absence of mitochondrial fragmentation. On the other hand, the DRP-1 levels were significantly elevated in the cells expressing mutant hCHCHD2 and which could be lowered by the p32-I treatment (Fig. 6B). Notably, the ability of p32 inhibition to lower DRP-1 levels in the two mutant hCHCHD2 expressing cells were found to be different and this is likely attributed to the nature of the different missense mutations.

In the mitochondria, energy in the form of adenosine-5’-triphosphate (ATP) is generated through electron transport and oxidative phosphorylation [61]. Hence fragmentation of the mitochondrial network will affect ATP production. We found that Hela cells expressing hCHCHD2 had lower ATP levels as compared to the control cell line expressing Myc. When incubated with the p32-inhibitor, the ATP levels of cells expressing mutant hCHCHD2 but not wildtype hCHCHD2 could be restored to near normal level (Fig. 6C). As mitochondria has role in controlling cell death [62], we checked the cell viability in these models and found that cell viability was reduced in the Hela cells expressing mutant CHCHD2 proteins and which could be restored by p32-I treatment (Fig. 6D).

### p32-I improves mitochondrial morphology and function of *hCHCHD2-Arg145Gln* NPCs and dopaminergic neurons and reduces α-synuclein level in the NPCs

**Fig. 7:**
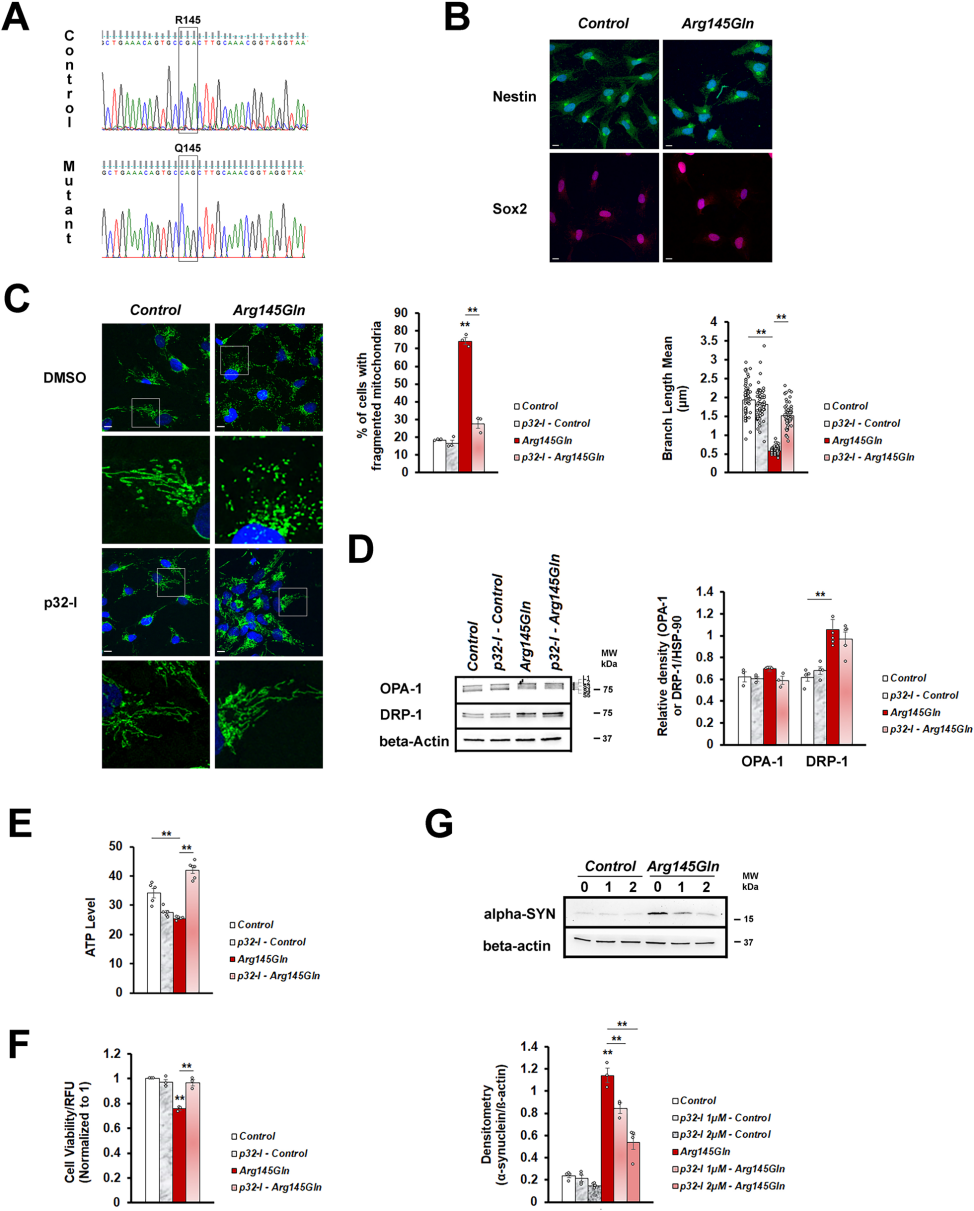
p32-inhibitor rescues mitochondrial morphological defects and functions and reduces α-synuclein expression of *CHCHD2-Arg145Gln* NPCs. **A** DNA sequence validation for the presence of *CHCHD2-Arg145Gln* mutation and its isogenic control. **B** Representative confocal images of immunoreactivity for Nestin (green), Sox2 (red) and DAPI (blue). Scale bar = 10 µm. **C** Representative confocal images of immunoreactivity for the mitochondrial marker (TOM20, green) and the nuclei marker (DAPI, blue) in the neural precursor cells. Normal mitochondria appear tubular in appearance as a sign of successful fusion and fragmented mitochondria appear as dots. Magnified regions are boxed. Scale bar = 10 µm. Quantification of fragmented mitochondria. The numbers of mitochondria analyzed for each group are between 279 to 355 (n = 3). Measurement of mitochondrial branch length. **D** Representative Western blot and quantitation of OPA-1 (n = 3) and DRP-1 (n = 4 for Control, p32-I – Control groups; n = 5 for Arg145Gln, p32-I – Arg145Gln groups). All forms of OPA-1 are indicated. Molecular weight markers are indicated in kDa. **E** Representative graph showing ATP levels in the *CHCHD2-Arg145Gln* line as compared to the Control, in the absence or presence of p32-inhibitor (n = 5 for all groups, except n = 6 for p32-I – Arg145Gln group). **F** Cell viability of *CHCHD2-Arg145Gln* NPC, in the absence or presence of p32-inhibitor (normalized to the control cells, n = 3). **G** Representative Western Blot showing α-synuclein expression and densitometric analysis. Genotypes are isogenic control cells (untreated, treated with 1 µM p32-I, treated with 2 µM p32-I) and *CHCHD2-Arg145Gln* cells (untreated, treated with 1 µM p32-I, treated with 2 µM p32-I). β-actin is the reference protein (n = 4 for all groups, except n = 3 for Arg145Gln, p32-I 1 µM – Arg145Gln groups). n = experimental repeats. Data are presented as mean ± SEM. Statistics used is One-way ANOVA followed by Bonferroni post hoc test. ^*^p < 0.05; ^**^p < 0.01

We further tested the effect of p32-inhibition using the isogenic line harboring *CHCHD2-Arg145Gln*. The *CHCHD2-Thr61Ile* mutant line was not generated due to the lack of specific gRNA sequence which is devoid of off-target activity. As the *CHCHD2* mutations are likely to be neo-morphic with gain of toxic function, we generated a homozygous *CHCHD2-Arg145Gln* mutant line so that the resulting mutant phenotypes would be strong enough for the p32-I to show clear rescue effects. To do this, the CRISPR genome editing technique was employed to generate mutant *CHCHD2-Arg145Gln* and its isogenic control lines from a human fibroblast cell line (Fig. 7A), followed by generation of iPSCs. The iPSCs were differentiated into neural precursor cells (NPCs) as shown by expression of the NPC markers (Nestin and Sox2, Fig. 7B). The NPCs harboring *CHCHD2-Arg145Gln* mutation had high degree of mitochondrial fragmentation (Fig. 7C), increased DRP-1 level (Fig. 7D), decreased ATP level (Fig. 7E) and reduced cell viability (Fig. 7F), which could be rescued by p32-I treatment. As α-synuclein accumulation and aggregation are pathological hallmarks of PD and had been observed in a number of PD models including the CHCHD2 knock-in mice model [63, 64], we investigated α-synuclein expression in the *CHCHD2-Arg145Gln* NPCs (Fig. 7G). We found that the NPCs harboring *CHCHD2-Arg145Gln* mutation had a marked increase in α-synuclein expression (Fig. 7G) when compared to the isogenic control line. To investigate if the different concentration of p32-I would have different effects on α-synuclein expression, we treated the NPCs with 1uM and 2uM of p32-I, both of which were tested to be non-toxic for cells expressing mutant hCHCHD2 (Fig. S1A). Interestingly, not only that the p32-I treatment could significantly reduce the α-synuclein protein level, but it did so in a dose dependent manner. More reduction of α-synuclein level was observed when the NPCs were treated with higher concentration of the p32-I (Fig. 7G).

**Fig. 8:**
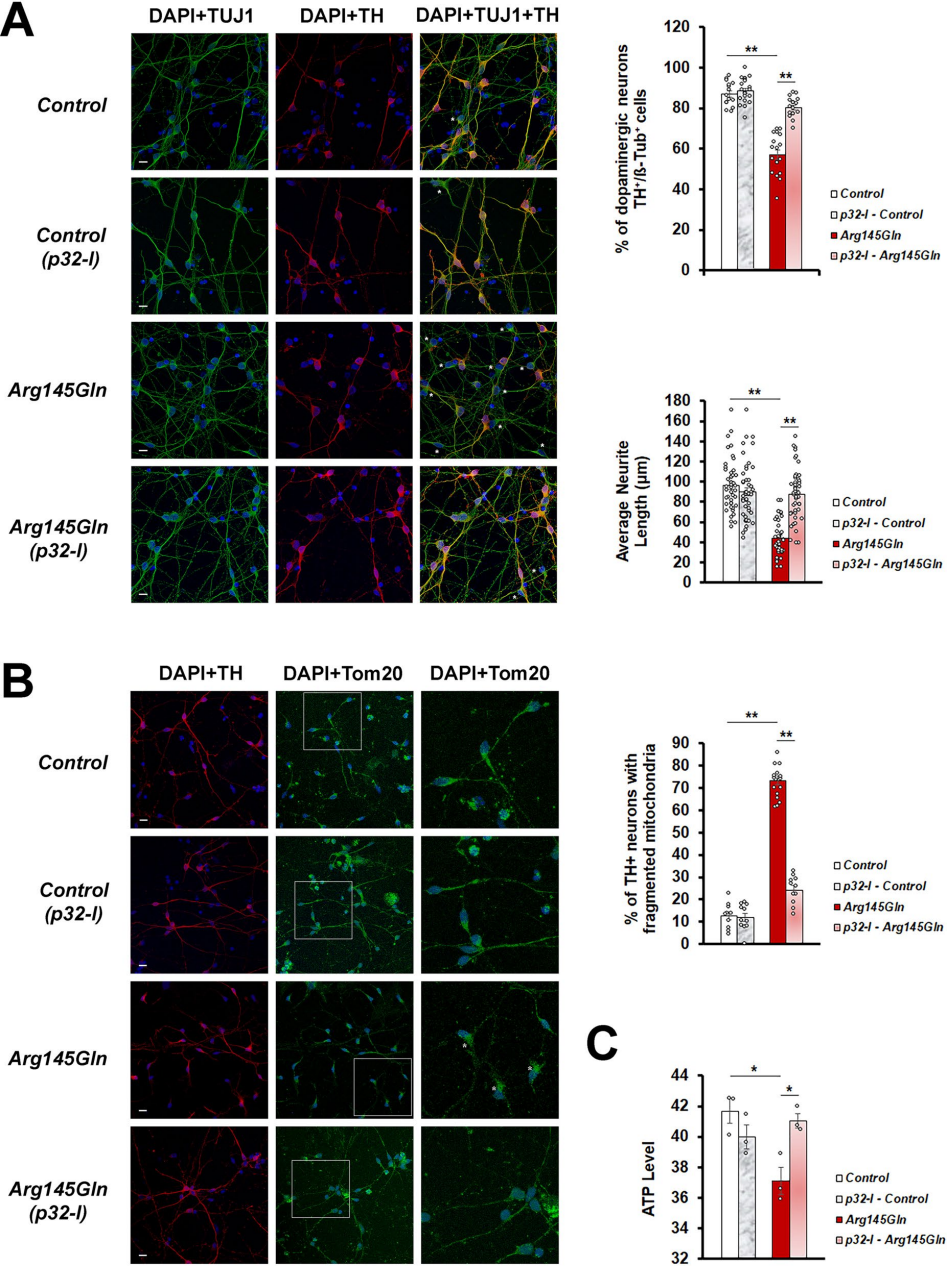
p32-inhibitor rescues dopaminergic fate, neurite length, mitochondrial morphology and function of *CHCHD2-Arg145Gln* neurons. **A** Representative confocal images of immunoreactivity for the dopaminergic neuronal marker (TH; red), the pan-neuronal marker (TUJ1; green) and the nuclei marker (DAPI; blue). Scale bar = 10 µm. Quantitation of TH expressing neurons (n = 3, each involving at least 100 neurons). Comparison of neurite length of *CHCHD2-Arg145Gln* neurons versus the Control neurons, in the absence or presence of p32-I. **B** Representative confocal images of immunoreactivity for the dopaminergic neuronal marker (TH; red), the mitochondrial marker (TOM20; green) and the nuclei marker (DAPI; blue). Dopaminergic neurons with normal mitochondria have tubular TOM20 staining in the neuronal cell bodies and their projections and those with abnormal mitochondria have fragmented mitochondria in the cell bodies and absence of or reduced TOM20 staining in the axonal projection. Magnified regions are boxed. Scale bar = 10 µm. Quantitation of the mitochondrial morphology (n = 3, each repeat involving at least 100 mitochondria). **C** Measurement of ATP levels in the *CHCHD2-Arg145Gln* neurons as compared to the Control neurons, in the absence or presence of p32-I (n = 3). In all graphs, statistics used is One-way ANOVA with Bonferroni post hoc test. n = experimental repeats. Data are presented as mean ± SEM. ^*^p < 0.05; ^**^p < 0.01

We also tested the rescue ability of the p32-I in the dopaminergic neurons which were differentiated from the NPCs. The *CHCHD2-Arg145Gln* neurons displayed lower DA differentiation efficiency as shown by fewer number of neurons expressing tyrosine hydroxylase (TH). These TH-expressing neurons also had shorter neurite extensions as compared to its isogenic control line (Fig. 8A). Treatment with the p32-I was able to restore DA differentiation efficiency and increase neurite length in these neurons (Fig. 8A). Higher number of TH-expressing *CHCHD2-Arg145Gln* neurons were found to contain fragmented mitochondria (Fig. 8B) and which was accompanied by lower ATP level (Fig. 8C). p32-I treatment was able to reduce mitochondrial fragmentation as well as restore ATP level in the *CHCHD2-Arg145Gln* neurons (Fig. 8B, C).

## Discussion

In this study, we investigated the functional interaction between CHCHD2 and p32 and identified p32 as a modulator of CHCHD2-linked PD phenotypes. We found that knockdown of p32 could mitigate all the PD-linked phenotypes in our *Drosophila hCHCHD2* models, possibly through down-regulation of the toxic mutant protein expression and mitigation of the downstream effects.

We found that dopamine levels decreased and H_2_O_2_ levels increased in the young hCHCHD2 transgenic *Drosophila* and at this stage the dopaminergic neuron degeneration and the locomotor impairment have not commenced [51]. Although CHCHD2-linked PD patients had been carrying the mutations since birth and similarly the *hCHCHD2* transgenic *Drosophila* had been expressing mutant hCHCHD2 since the first day of adult development, the PD-like characteristics appeared to be late-onset. This suggests that age and aging associated events play important roles in disease development and progression. We demonstrated that p32 knockdown increased the dopamine levels as well as reduced oxidative stress in young *Drosophila* expressing mutant hCHCHD2 and these effects could be correlated with the rescue of dopaminergic neuron loss and the locomotor impairment in older flies, suggesting that interception of phenotypes at early stage can slower phenotypic progression. Among the known roles of dopamine is to control motor function. In PD patients, dopaminergic dysfunction often precedes degeneration of nigral cells and patients only become symptomatic when the majority of the nigra dopaminergic neurons have been lost [65]. Hence, detection of dopamine level or dopamine function through brain imaging offers a way to predict clinical outcomes in PD patients and to intervene early before the irreversible DA neuron degeneration takes place.

We found that p32 knockdown could modulate the expression of hCHCHD2 mutant proteins in both the *Drosophila* and the cell models. In *Drosophila*, although wildtype and mutant hCHCHD2 proteins are similarly expressed at early stage of *Drosophila* development [51], the expression of wildtype hCHCHD2 increases more with age as compared to those of the mutant hCHCHD2 proteins. Notably, although p32-knockdown could lower mutant hCHCHD2 protein levels in both the *Drosophila* and the Hela cells, it was not effective in lowering the wildtype hCHCHD2 level. Continuous high level of WT hCHCHD2 could have caused toxicity in combination with certain age-related factors such as presence of higher oxidative stress in older *Drosophila* [66], hence the worsened phenotypes in flies that concurrently expressed *p32* RNAi and wild type hCHCHD2. The different effects of p32 knockdown may also be explained by cell context specificity. Differential effects of p32 knockdown have also been reported to be dependent on the nature of the stress imposed as well as the level of the particular stress [67]. The ability of p32 knockdown to reduce mutant hCHCHD2 protein levels might not only have resulted in lower toxicity but also triggered other protective mechanism. Similar phenomenon whereby mutant cells are more able to respond to oxidative stress with age than WT cells has been reported for the cultured cortical *Afg3l2*-KO neurons. In the presence of acute stress, *Afg3l2*-KO neurons but not the WT neurons activated a protective mechanism that involved massive upregulation of a mitochondrial antioxidant, Peroxiredoxin-3 (Prx3) [68]. Interestingly, we have previously shown that *Drosophila* expressing mutant hCHCHD2 were more responsive towards treatment with Ebselen (a peroxiredoxin mimic) [69] than the transgenic *Drosophila* expressing WT hCHCHD2 or the risk variant, Pro2Leu [51]. How p32 inhibition resulted in mitigation of the PD-like characteristics in *Drosophila* mutant hCHCHD2 models remains to be investigated. p32 inhibition could have triggered similar protective mechanisms which attenuated the mutant phenotypes.

The p32 protein is known to have both pro-apoptotic and proliferative roles. The p32-inhibitor was able to rescue the neurodegeneration phenotypes in our disease models. This is intriguing considering that cancer and neurodegeneration are two diseases of opposing characteristics, with cancer cells undergoing uncontrolled cell division and degenerating neurons undergoing premature cell death. An example of a protein with both tumour-suppressor and neuro-protective functions is Parkin [70]. The neuro-protective role of Parkin is linked to its ubiquitin ligase activity of the ubiquitin–proteasome system (UPS), which is responsible for intracellular protein degradation [70, 71]. Parkin also triggers elimination of abnormal mitochondria through mitophagy and hence preventing accumulation of abnormal protein aggregates and dysfunctional mitochondria which causes degeneration of neurons [71]. Parkin is also known for its tumor suppressor and anti-metastasis properties. HIF-1 (hypoxia-inducible factor 1) is a protein which plays a role in cancer metastasis and binding of Parkin to HIF-1 targets HIF-1 for ubiquitination and degradation and thus prevents metastasis of breast cancer cells [72]. These observations suggest the presence of cell context specificity in disease development and drug response.

At the moment, we do not have a clear explanation of how p32-knockdown lowered mutant but not wildtype hCHCHD2 levels. One possible explanation could be due to cell context specific function of p32. p32 had been reported to be one of the interacting partners of parkin, although it is not a parkin substrate [73]. In the study, p32 was shown to degrade parkin through the autophagy pathway but it did not affect the autoubiquitination ability of parkin [73]. Knockdown of p32 was found to increase parkin protein level and cause mitochondrial fragmentation [35, 73]. Whether or not p32 exerted differential effects in regulating hCHCHD2 levels through parkin in our *Drosophila* and Hela overexpression models need further investigation. p32-inhibition could cause an increase in parkin protein level and its ubiquitination activity, leading to the decrease of mutant hCHCHD2 levels through autophagic clearance. On the other hand, p32-inhibition could cause the opposite effects in the models overexpressing wildtype hCHCHD2. Nevertheless, the reports that p32-knockdown caused mitochondrial fragmentation provide one explanation for the observed phenotypic and mitochondrial abnormality in the control and the *Drosophila* overexpressing wildtype hCHCHD2, in the presence of p32-inhibition. Despite the discovery of p32 and parkin interaction and the known role of parkin in PD pathogenesis, the role of p32 in PD is still unclear as mutations in p32 have so far not been reported in PD patients.

Ikeda and colleagues reported that brain sample from the Japanese patient carrying *hCHCHD2-Thr61Ile* mutation showed α-synuclein pathology with Lewy bodies (LBs) in a few regions of the brain [74]. Fan and colleagues also reported that *hCHCHD2-Thr61Ile* knock-in mice and iPSC-derived dopaminergic neurons harboring *hCHCHD2-Thr61Ile* had accumulation and aggregation of α-synuclein in the brain, which was accompanied by presence of abnormal mitochondria [64]. Our isogenic line harboring *hCHCHD2-Arg145Gln* contained high level of α-synuclein, and interestingly, the p32-I was able to decrease the α-synuclein level in a dose dependent manner. Previously, a number of reports have shown that expression of toxic proteins or protein aggregates could trigger mitochondrial fragmentation phenotype which was accompanied by elevated DRP-1 level [75]. Although the status of DRP-1 levels in post-mortem brains of patients having CHCHD2 mutations remained to be investigated, our observations in *Drosophila* and cell models of *hCHCHD2-Thr61Ile* suggest that mitochondrial fragmentation linked to elevated DRP-1 level likely contributes to the pathogenesis of CHCHD2-linked PD. Despite the non-significant effects of p32-I in lowering DRP-1 levels in transgenic *Drosophila* expressing *hCHCHD2-Arg145Gln* and in isogenic line harboring *hCHCHD2-Arg145Gln*, p32-I was able to correct mitochondrial morphology and restore ATP production, suggesting that p32-I could rescue disease phenotypes via mechanisms other than through lowering the DRP-1 levels. Alternatively, since *hCHCHD2-Thr61Ile* and *hCHCHD2- Arg145Gln* are two different missense mutations which are likely to possess different properties, these results can also mean that the two mutant lines require different low threshold level of DRP-1 expression for the rescue of mitochondrial morphology and function.

## Conclusion

In summary, our in vivo and in vitro studies suggest that expression of mutant hCHCHD2 likely results in toxicity leading to the observed phenotypes including high degree of mitochondrial fragmentation/mitochondrial dysfunction and accumulation of α-synuclein. We highlight for the first time that genetic knockdown of p32 and pharmacological p32 inhibition can ameliorate disease phenotypes in hCHCHD2-linked PD models likely through improvement of mitochondria morphology/function and reduction of α-synuclein level. Our study suggests that inhibition of the p32 pathway can provide a novel therapeutic approach for CHCHD2-linked PD.

### Supplementary Information

**Additional file 1: Figure S1.** Toxicity testing and validation of the p32-inhibitor. **A** A range of p32-inhibitor concentrations from 0 to 250 µM were tested on the Hela cells expressing CHCHD2-WT-MYC, CHCHD2-Thr61Ile-MYC and the Control cells expressing MYC alone. Note that 2.5 µM, 5 µM and 7.5 µM of p32-I are more toxic to the Hela cells expressing CHCHD2-WT-MYC as compared to the Control (n = 3, One-way ANOVA with Bonferroni post hoc test). Hence, 1 µM p32-I is chosen for cell treatment. **B** Representative Western blots showing effectiveness of 1 µM p32-I. Data are presented as mean ± SEM of three independent experiments. ^*^p < 0.05; ^**^p < 0.01.

## Data Availability

All data generated and analyzed during this study are included in this article and its Additional file 1. The data and materials used in this study are available upon reasonable requests to the corresponding author.

## Abbreviations

PD: Parkinson’s disease
*SNCA*: *alpha-synuclein* (Gene)
*LRRK2*: *leucine-rich repeat kinase 2*
*PARK2*: *Parkinson juvenile disease protein 2/Parkin*
*PARK7*: *Parkinson disease protein 7/DJ-1*
PINK1: PTEN-induced kinase 1
CHCHD2: Coiled-coil-helix-coiled-coil-helix domain containing 2
ATP: Adenosine triphosphate
OPA-1: Optic atrophy-1
DRP-1: Dynamin-related protein-1
CRISPR: Clustered regularly interspaced short palindromic repeats
Cas9: CRISPR-associated protein 9
iPSC: Induced pluripotent stem cells
NSCs: Neural stem cells
NPCs: Neural progenitor cells
p32-I: p32-inhibitor
RT: Room temperature
TH: Tyrosine hydroxylase
HPLC: High performance liquid chromatography
Co-IP: Co-immunoprecipitation
TEM: Transmission electron microscopy
SEM: Standard error of the mean
RNAi: RNA interference
Prx-3: Peroxiredoxin-3
ALS/FTD: Amyotrophic lateral sclerosis/frontotemporal dementia
