# Supplementary figures and images for: Genetic and pharmacologic p32-inhibition rescue CHCHD2-linked Parkinson’s disease phenotypes in vivo and in cell models
Source: J Biomed Sci. 2024 Feb 23;31:24. doi: 10.1186/s12929-024-01010-z (PMC10893700; doi:10.1186/s12929-024-01010-z)

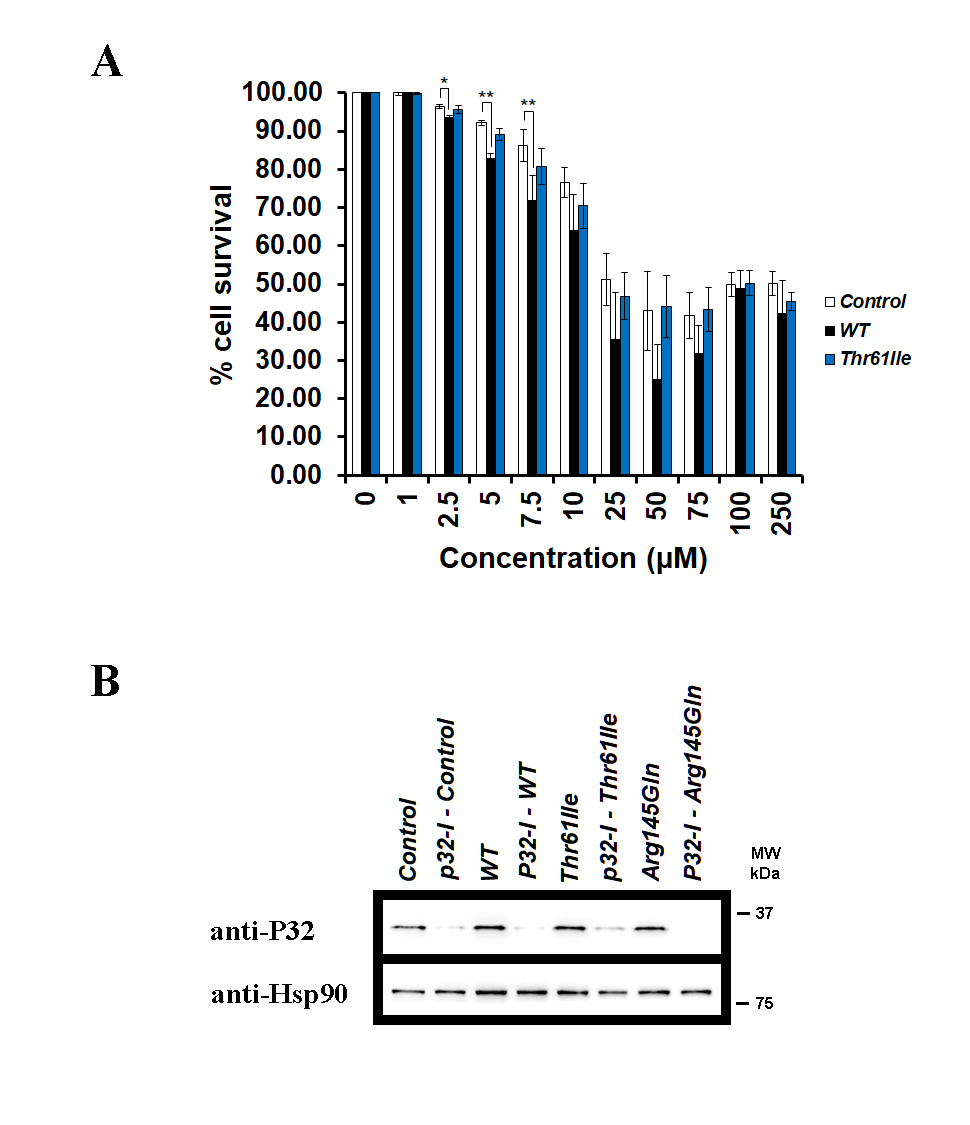

Supplement: Supplementary file 1 — Additional file 1: Figure S1. Toxicity testing and validation of the p32-inhibitor. A A range of p32-inhibitor concentrations from 0 to 250 µM were tested on the Hela cells expressing CHCHD2-WT-MYC, CHCHD2-Thr61Ile-MYC and the Control cells expressing MYC alone. Note that 2.5 µM, 5 µM and 7.5 µM of p32-I are more toxic to the Hela cells expressing CHCHD2-WT-MYC as compared to the Control (n = 3, One-way ANOVA with Bonferroni post hoc test). Hence, 1 µM p32-I is chosen for cell treatment. B Representative Western blots showing effectiveness of 1 µM p32-I. Data are presented as mean ± SEM of three independent experiments. *p < 0.05; **p < 0.01. [file 12929_2024_1010_MOESM1_ESM.jpg]
